# Clinical yield and operational cost-threshold analysis of HPV self-sampling send-outs for long-term non-attenders in cervical cancer screening: a population-based, real-world, registry study in Sweden

**DOI:** 10.1136/bmjopen-2026-125034

**Published:** 2026-10-22

**Authors:** Jasmine Fridljung, Lovisa Bergengren, Gabriella Lillsunde-Larsson, Linda Ryen

**Affiliations:** 1 University Health Care Research Centre, Faculty of Medicine and Health, Örebro University, Örebro, Sweden; 2 Department of Women’s Health, Faculty of Medicine and Health, Örebro University, Örebro, Sweden; 3 Department of Laboratory Medicine, Faculty of Medicine and Health, Örebro University, Örebro, Sweden; 4 School of Health Sciences, Örebro University, Örebro, Sweden

**Keywords:** Health economics, HPV Infection, Mass Screening, Public health, Gynaecological oncology

## Abstract

**Abstract:**

**Objectives:**

To evaluate the clinical yield and operational costs of a targeted ‘send-to-all’ human papillomavirus (HPV) self-sampling strategy for long-term non-attenders in cervical cancer screening.

**Design:**

Population-based, real-world observational registry study.

**Setting:**

The regional organised cervical cancer screening programme in Region Örebro County, Sweden.

**Participants:**

A total of 9123 women identified as long-term non-attenders. Long-term non-attendance was defined as having no recorded cervical screening test for at least one routine screening interval plus an additional 4 years.

**Interventions:**

A total of 11 283 vaginal HPV self-sampling kits were dispatched via direct mail to the registered home addresses of all 9123 eligible women between 2022 and 2024.

**Primary and secondary outcome measures:**

Clinical yield was evaluated using the number of tests analysed (NTA) and number of kits dispatched (NKD) to detect one case of high-grade squamous intraepithelial lesion or worse (HSIL+). Resource utilisation was measured as the average cost per screened woman/detected HSIL+, and the economic break-even point against standard letter invitations was established using a deterministic threshold analysis.

**Results:**

Of the 11 283 self-sampling kits dispatched, 1826 kits were returned and analysed. This direct response yielded 27 HSIL+ cases (NTA: 68; NKD: 418), at an average cost of 2031 SEK (€183) per screened woman and 134 103 SEK (€12,114) per histologically confirmed HSIL+ case. Incorporating an additional 190 long-term non-attenders who underwent clinical screening within 1 year of dispatch yielded a total of 32 HSIL+ cases. This cumulative response decreased the NTA to 63 and the NKD to 353, with an average cost of 2045 SEK (€185) per screened woman and 126 068 SEK (€11 388) per HSIL+ case. To match the cost per screened woman of the self-sampling strategy, standard letter invitations must achieve a participation rate of 9.5% (based on kit returns alone) or 6.4% (based on cumulative participation).

**Conclusions:**

Direct mailing of HPV self-sampling kits effectively identifies high-grade lesions in a hard-to-reach population. From an operational perspective, previous research indicates that participation from standard letters rarely reaches the calculated 6.4%–9.5% break-even points, suggesting self-sampling is a resource-efficient initial outreach strategy for long-term non-attenders. However, full economic evaluations incorporating long-term health outcomes are required to establish comprehensive cost-effectiveness.

**STRENGTHS AND LIMITATIONS OF THIS STUDY**

- The population-based, real-world design evaluates the routine implementation of a targeted self-sampling strategy across an entire healthcare region.
- Using a deterministic threshold analysis provides healthcare planners with operational break-even points.
- The methodology includes an exploratory upper-bound scenario to capture additional clinical attendances that occurred following the kit dispatch. The threshold analysis is limited to the primary screening phase and does not account for downstream resource allocation.
- As registry data lack clinical indications, some attendances may be symptom-driven rather than a response to the intervention.

## Introduction

 Cervical cancer remains a major global health burden, ranking as the fourth most common cancer among women worldwide. In response, the WHO has launched a global strategy to accelerate the elimination of the disease as a public health problem.^1^ In contrast to the high global incidence, Sweden has a relatively low burden, largely attributable to a long-standing and successfully organised screening programme.^2^ However, despite high overall participation, a concerning disease burden remains, with an incidence rate still above the elimination target of fewer than 4 cases per 1 00 000 women per year.^3^ Participation is a key determinant of screening effectiveness. Even with annual screening invitations, a subset of eligible women does not attend. This group is clinically important because long-term non-attendance is associated with a higher burden of cervical high-grade abnormalities and cancer compared with women who participate as recommended.^4 5^ The traditional model, inviting all women in the same manner regardless of their risk profile, has proven insufficient to reach this final, hard-to-reach fraction from which many of the cancer cases originate.^3^ Consequently, current preventive efforts are focused on refining the programme by identifying and reaching the remaining under-screened cohorts.^4^

The transition to primary human papillomavirus (HPV)-based cervical cancer screening has enabled the use of self-collected vaginal samples for HPV screening, which can be taken at home and analysed within the organised programme.^6 7^ Sending HPV self-sampling kits by mail has emerged as a strategy to overcome barriers to clinical screening. Randomised trials and meta-analyses show that offering self-sampling, particularly via direct mailing of kits, can increase participation compared with standard letter invitations.^5 8–10^ As a result, in several Swedish regions, HPV self-sampling is now used as a strategy to reach women who have not attended screening after standard invitations.^3 6 7^

As these interventions transition from trials to routine implementation, it becomes essential to evaluate their real-world clinical yield and resource utilisation. Screening programmes involve large populations and repeated testing, so even modest changes in participation, test positivity or follow-up pathways could have implications for healthcare resource allocation. In our specific setting, the direct mailing of HPV self-sampling kits (a ‘send-to-all’ approach) has been implemented as an addition to the standard invitation letters for long-term non-attenders. The fundamental operational question for healthcare providers, which this study seeks to address, is whether this regional dispatch successfully leads to more long-term non-attenders entering the system and generating a screening result.

Evaluating the cost-effectiveness of this real-world strategy is methodologically challenging due to a lack of historical control data for this specific, hard-to-reach cohort. In health economics, resource allocation is typically guided by an incremental cost-effectiveness ratio (ICER), a metric that quantifies the additional cost required to achieve one additional unit of health benefit (such as a life-year gained or a detected precancerous lesion) when comparing several interventions. However, because this intervention was rolled out as a routine implementation, and standard administrative systems rarely stratify historical attendance rates by prior screening history, establishing a reliable baseline for this specific subgroup is difficult. A traditional full-pathway ICER would therefore be heavily reliant on assumptions. To overcome this, we applied an alternative framework focusing on direct resource utilisation and operational effectiveness indicators specifically, the number of tests analysed (NTA) and number of kits dispatched (NKD) to detect one additional case of high-grade squamous intraepithelial lesion or worse (HSIL+). Furthermore, we conducted a threshold analysis to determine the economic break-even point compared with the standard letter invitation strategy.

## Method

### Study population

This study analysed the ongoing routine screening strategy for long-term non-attenders implemented in Region Örebro County, Sweden. The study population consisted of women defined as long-term non-attenders, meaning they had no recorded cervical cytology or HPV test for at least one screening interval (5–7 years depending on age) + an additional 4 years.^6^ Between 1 January 2022 and 31 December 2024, a total of 11 283 HPV self-sampling kits (the vaginal Aptima Multitest Swab Specimen Collection Kit) were automatically mailed to the registered home addresses of 9123 unique eligible women. Professional samples and returned self-samples were tested for high-risk HPV using the Aptima HPV assay (Hologic, Marlborough, MA, US). All HPV-positive samples subsequently underwent genotyping using the Allplex HPV HR Detection assay (Seegene, Seoul, Korea). Women with positive screening results were sent an invitation for a clinical follow-up, which was due within 2–3 months. Follow-up data were included up until 30 April 2026. During this period, new standard routine screening invitations may have been distributed concurrently by the general screening programme.

To capture direct and indirect effects of the intervention, the study population was stratified into two cohorts:

The direct response cohort: Includes women who returned the self-sampling kit. For the calculations of clinical yield and resource utilisation, follow-up was included if it was initiated within 1 year of the self-sampling screening result.

The cumulative response cohort: This cohort includes the direct response cohort as well as women who did not return the dispatched kit but instead attended clinical screening within 1 year of its dispatch. By capturing these additional clinical attendances, this cohort serves as an exploratory upper-bound scenario of the potential secondary responses to the outreach strategy in a real-world clinical setting.^9^

The complete participation and clinical pathway is illustrated in figure 1.

**Figure 1:**
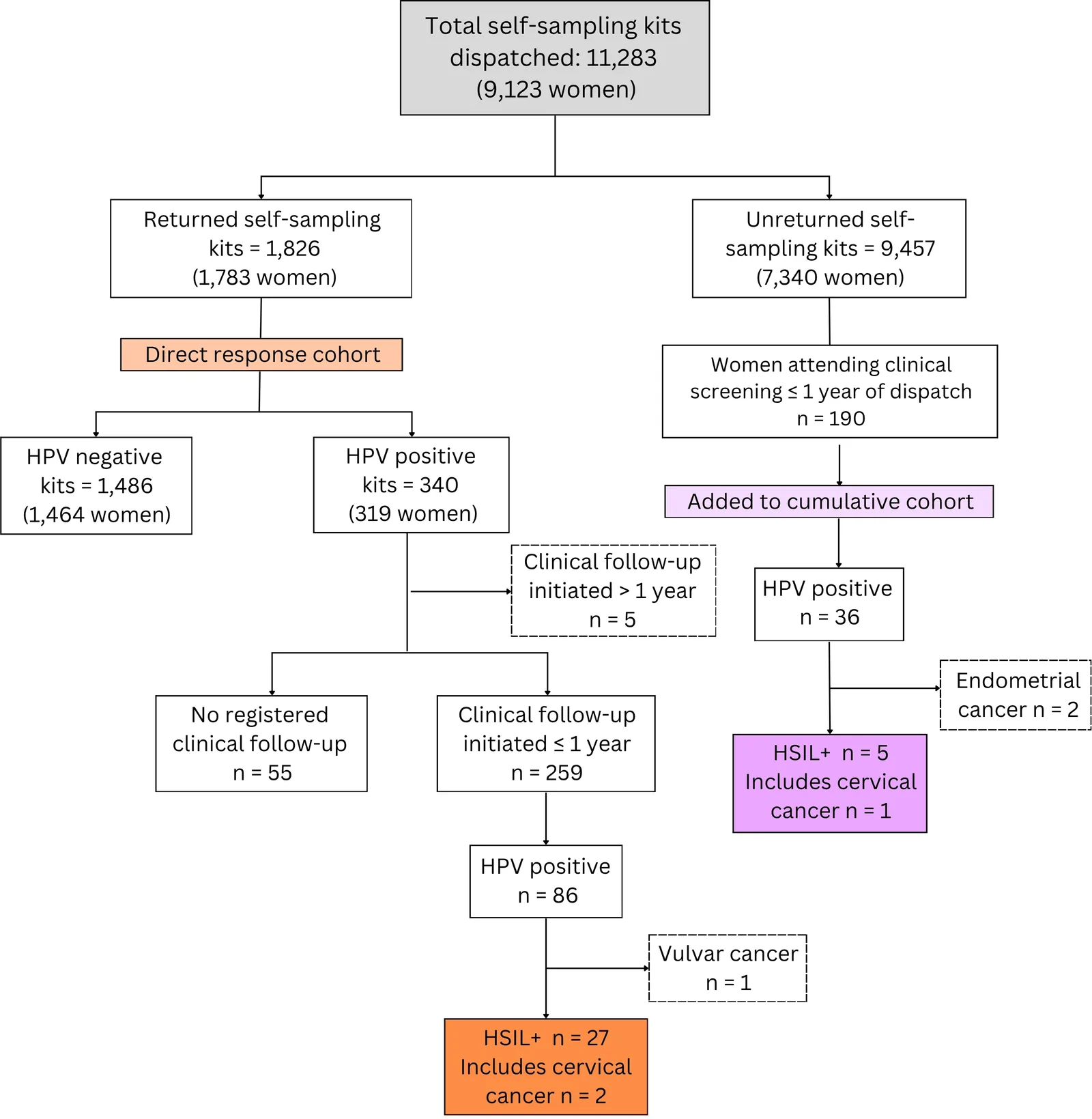
Flow chart. A total of five individuals with delayed follow-up (>1 year) and three non-cervical cancers were excluded from the clinical yield and resource use calculations (indicated by dashed boxes). HPV, human papillomavirus; HSIL+, high-grade squamous intraepithelial lesion or worse.

### Study design

The clinical yield and resource utilisation of the HPV self-sampling strategy were assessed using a two-part framework. All calculations were performed using Microsoft Excel (V.2604, Microsoft 365).

#### Clinical yield and resource utilisation

The analysis was restricted to the initial clinical visit occurring within 1 year of a returned self-test or initial dispatch. These visits comprised either follow-up appointments (midwife visits or colposcopy) following a positive self-test or clinical screening attendances (midwife visits or colposcopy) for women who did not return the self-sampling kit (figure 1). For each individual, data were included up to the point of diagnosis; downstream treatment interventions and subsequent routine screening tests were excluded, as these women no longer met the criteria for long-term non-attenders. However, to accurately reflect the overall response to the dispatch strategy, all dispatched and returned self-sampling kits were included. Clinical effectiveness and resource utilisation were evaluated using the following indicators:

*Number of tests analysed to detect one HSIL+*


(1) $$NTA_{detect}=\;\frac{N_{screened}}{N_{HSIL+detected}}$$

${NTA}_{detect}$ (Equation 1) was calculated as the number of analysed self-sampling kits divided by the total number of histologically confirmed HSIL+ cases. This reflects the HSIL+ prevalence within the responding cohort. For the cumulative cohort, the numerator is the combined total of analysed self-sampling kits and additional clinical screening visits.

*Number of kits dispatched to detect one HSIL*+


(2) $$NKD_{detect}=\;\frac{N_{dispatched}}{N_{HSIL+detected}}$$

${NKD}_{detect}$ (Equation 2) was calculated as the total number of dispatched kits divided by the number of histologically confirmed HSIL+ cases. This quantifies the operational effectiveness of the outreach strategy by accounting for non-participation.

To avoid confusion with the traditional metrics ‘number needed to screen’ and ‘number needed to invite’ used in longitudinal mortality studies which define them as the reciprocal of the absolute risk reduction to prevent a single death,^11^ this study uses NTA and NKD to specifically evaluate operational and diagnostic yield.^12 13^

#### Resource utilisation

Expressed as the average cost per successfully screened woman and as the average cost per histologically confirmed HSIL+.

#### Deterministic threshold analysis (screening phase)

A deterministic threshold analysis (DTA) was performed to establish the operational break-even point compared with the standard strategy. In this context, the standard strategy consists of a mailed standard invitation letter assigning a scheduled appointment, followed by a midwife-led clinical screening visit and subsequent HPV analysis. The DTA excludes downstream clinical triage and follow-up (eg, genotyping of positive HPV-tests or histological examinations) and focuses solely on the screening phase (eg, an analysed self-sampling test or a clinical screening visit). A bottom-up micro-costing approach was applied from a healthcare provider perspective, where the cost burden of all non-returned kits was absorbed by the participating cohort.

DTA is a form of one-way sensitivity analysis used to determine the parameter value at which the conclusion of an economic model changes.^14–16^ While the baseline unit costs for sending standard invitations ($C_{invite}$, set to 23 SEK) and conducting midwife-led screening visits ($C_{test}$, set to 388 SEK) are predictable within the regional administrative systems, the participation rate (*p*) among long-term non-attenders in response to standard invitations remains uncertain. Therefore, a DTA was performed to establish the operational break-even point at which the standard strategy would achieve cost-neutrality per screened woman compared with the current targeted self-sampling strategy. The underlying formula was derived by setting the average cost to obtain an analysed screening test as equal for both strategies and solving for the required response rate (*p*) (see online supplemental appendix A). The threshold analysis was conducted across the two cohorts:


(3) $$\begin{array}{rl} p & =\frac{C_{invite}}{AC_{self\_direct}-C_{test}} \end{array}$$

(4) $$\begin{array}{rl} p & =\frac{C_{invite}}{AC_{self\_cumulative}-C_{test}} \end{array}$$

Where $C_{invite}$ is the unit cost of a standard invitation, ${AC}_{self\_direct}$ is the observed average total cost per successfully screened woman in the direct response cohort (Equation 3), ${AC}_{self\_cumulative}$ is the total cost per screened woman in the cumulative response cohort (Equation 4) and $C_{test}$ is the unit cost of a standard midwife-led screening visit (including consumables and laboratory analysis).

### Data

Data were extracted for all self-sampling kits dispatched between 1 January 2022 and 31 December 2024. Follow-up data were included up until 30 April 2026. Data on dispatched kits, kit return status, HPV analysis results and follow**-**ups were retrieved from the regional laboratory system Sympathy. Data on resource use and unit costs were obtained directly from the regional administrative systems and cost per patient database.

During the study period, standard routine screening invitations may have been distributed concurrently by the general screening programme. To account for these concurrent invitations, a proportional cost allocation was applied. Because exact individual dispatch timestamps vary across the registry period, a single standard letter cost (23 SEK) was assigned to the sub-cohort of absolute non-attenders (n=7150; ie, women who neither returned a self-sampling kit nor attended clinical screening). This approach provides an approximation of the concurrent administrative burden.

All costs were calculated in Swedish Krona (SEK) and are summarised in table 1 (for a detailed breakdown of mailing, laboratory and workforce unit costs, see online supplemental appendix B). For international comparability, the primary economic outcomes are also presented in Euros (€), converted using the official 2025 annual average exchange rate provided by the Swedish Central Bank (1 € = 11.07 SEK).

**Table 1:** Resource utilisation and total costs for the direct and cumulative response cohorts

	Unit cost	Direct cohort		Cumulative cohort	
Resource component	(SEK)	Quantity (n)	Cost (SEK)	Quantity (n)	Cost (SEK)
Dispatched self-sampling kits	45	11 283	507 735	11 283	507 735
Concurrent standard invitations	23	7150	164 450	7150	164 450
HPV analysis of returned kits	246	1826	449 196	1826	449 196
Midwife screening visits (incl. consumables and HPV analysis)	388	22	8536	169	65 572
Colposcopies (incl. HPV-analysis and cytology)	7823	234	1 830 582	272	2 127 856
Genotyping of HPV-positive tests	915	424	387 960	458	419 070
Histopathological analysis	1472	185	272 320	204	300 288
Total cost			3 620 779		4 034 167

All costs are expressed in Swedish Krona (SEK) at the 2025 price level, with the exception of unit costs for colposcopies which are based on 2024 data. The number of colposcopies is a derived estimate based on registered referrals for vaginal cytology. Values in the cumulative cohort include the resource utilisation from the direct cohort. To account for concurrent standard invitations, a single letter cost was allocated to the 7150 absolute non-attenders (women who neither returned a kit nor attended clinical screening).

HPV, human papillomavirus.

## Results

### Clinical yield and resource utilisation

During the study period, a total of 11 283 HPV self-sampling kits were dispatched to 9123 eligible long-term non-attenders. Of these, 1783 unique women returned kits that were successfully analysed (figure 1). Among the screened women in this direct response cohort, 319 individuals tested positive for HPV. The majority attended clinical follow-up within 1 year, leading to the detection of 27 HSIL+ cases, including 2 cervical cancer cases. A small subset of the HPV-positive women either lacked registered follow-up or experienced delays exceeding 1 year.

In the cumulative response cohort, which included women who did not return the kit but attended a clinical screening within 1 year of dispatch, an additional 190 women were screened. Of these, 36 were HPV-positive, yielding an additional 5 HSIL+ cases, including 1 cervical cancer case. A breakdown of participation rates, clinical follow-up adherence and operational yield for both cohorts is provided in table 2.

**Table 2:** Clinical yield and resource utilisation

Parameter	Direct response cohort	Cumulative response cohort
Outreach and participation		
Total dispatched kits (n)	11 283	11 283
Returned kits (n)	1826	1826
Kit return rate (%, 95% CI)	16.2 (15.5 to 16.9)	16.2 (15.5 to 16.9)
Screened women (n)	1783	1973
Participation rate (%, 95% CI)	19.5 (18.7 to 20.4)	21.6 (20.8 to 22.5)
Clinical findings and follow-up		
HPV-positive women (n)	319	355
HPV positivity rate (%, 95% CI)	17.9 (16.2 to 19.7)	18.0 (16.4 to 19.7)
No registered clinical follow-up (n, %)	55 (17.2%)	–
Delayed follow-up >1 year (n, %)	5 (1.6%)	
Confirmed HSIL+ cases (n)	27	32
Operational and economic outcomes		
NTA_detect_	68	63
NKD_detect_	418	353
Average cost per screened woman (SEK)	2031	2045
Average cost per detected HSIL+ (SEK)	134 103	126 068

The cumulative response cohort includes an additional 190 women who attended clinical screening within 1 year of the kit dispatch. Average costs are derived from the total operational costs of the full clinical pathway (including both the screening and clinical follow-up phases) and are expressed in Swedish Krona.

HPV, human papillomavirus; HSIL+, high-grade squamous intraepithelial lesion or worse; NKD, number of kits dispatched; NTA, number of tests analysed; SEK, Swedish Krona.

#### The direct response cohort

Within the direct response cohort, the strategy demonstrated a NTA_detect_ of 68, indicating that 68 self-sampling kits needed to be analysed to detect 1 case of HSIL+. When accounting for all dispatched kits, the NKD_detect_ was 418 per confirmed HSIL+ case (table 2). Regarding resource utilisation (including the burden of unreturned kits), the observed average cost per screened woman was 2031SEK (€183) and the observed average cost to detect one additional HSIL+ case was calculated at 134 103 SEK (€12 114).

#### The cumulative response cohort

When incorporating clinical attendances, the diagnostic yield resulted in a NTA_detect_ of 63 and a NKD_detect_ of 353 per confirmed HSIL+ case (table 2). In this cumulative scenario, the economic burden of the unreturned kits was absorbed by a larger total pool of screened women, increasing the cost per successfully screened woman only slightly to 2045 SEK (€185). The inclusion of the additional high-grade lesions detected in this cohort decreased the average cost per detected HSIL+ case to 126 068 SEK (€11,388).

### Threshold analysis (screening phase)

#### The direct response cohort

Focusing solely on the screening phase of the direct response cohort, the total cost includes the administrative dispatch of all kits and the primary laboratory analysis of the 1826 returned tests, excluding any downstream diagnostic procedures. This total cost amounted to 1 121 381 SEK (€101 299). To accurately reflect the cost per screened individual, this total cost was divided by the 1783 uniquely screened women, absorbing the costs of duplicate tests. This yielded an average cost of 629 SEK (€57) per successfully screened woman (${AC}_{self\_direct}$). Applying the deterministic threshold formula, the unit cost of a standard annual invitation letter ($C_{invite}$) was set at 23 SEK (€2), and the unit cost of a standard clinical screening visit ($C_{test}$) was set at 388 SEK (€35).


$$\begin{array}{r} p=\;\frac{23}{629-388}=9.5\% \end{array}$$

Applying the parameters to Equation 3 yielded an operational break-even point of 9.5%. This implies that a standard letter invitation strategy, when directed at this specific demographic of long-term non-attenders, would need to reach a participation rate of 9.5% to equal the screening-phase cost per screened woman of the targeted self-sampling strategy (figure 2).

**Figure 2:**
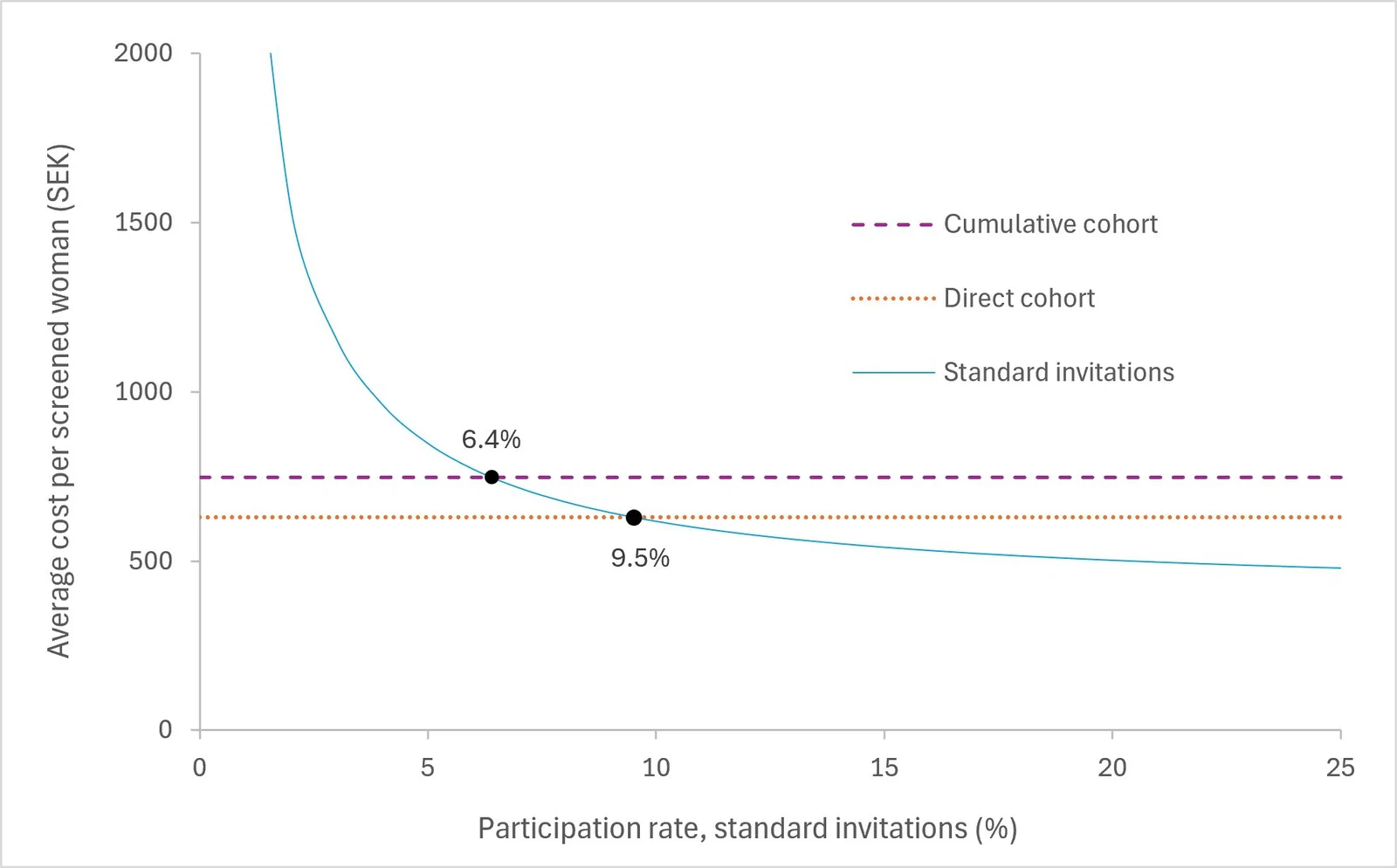
Deterministic threshold analysis. The graph illustrates the operational break-even points by comparing the average cost per screened woman using the self-sampling strategy against simulated participation rates for standard clinical invitations. The x-axis represents the hypothetical participation rate for the standard letter invitation strategy (solid line). The horizontal lines represent the observed, fixed average costs for the targeted self-sampling strategy. This analysis includes only the costs associated with the primary screening phase. Costs are based on the direct cohort (women who returned the dispatched self-sampling kit; dotted line) and the cumulative cohort (capturing the overall response, including also clinical screening attendance within 1 year; dashed line). The black dots indicate the break-even points (6.4% and 9.5%) at which standard letter invitations achieve cost-neutrality per screened woman compared with the self-sampling cohorts. SEK, Swedish Krona.

#### The cumulative response cohort

Expanding the threshold analysis to capture the broader impact increases the total cost of the screening phase to 1 475 691SEK (€133 305). This incorporates both the initial self-sampling outreach, the cost of analysed kits and the costs associated with the 190 clinical screening visits, comprising a mix of midwife-led HPV-screenings and physician-led colposcopies (including their respective initial HPV and cytology analyses). Downstream diagnostic triage and follow-ups resulting from these visits, such as HPV genotyping and histopathological analyses, were excluded to maintain a strict screening phase perspective. To determine the adjusted average cost per screened woman $({AC}_{self\_cumulative}$, this total was divided by the combined 1973 uniquely screened individuals (1783 from the direct response and 190 additional clinical screenings). This yielded an adjusted average cost of 748 SEK (€68) per successfully screened woman. Applying the same deterministic threshold formula, with the standard invitation unit cost ($C_{inv}$) remaining at 23 SEK (€2) and the standard clinical screening visit cost ($C_{test}$) at 388 SEK (€35):


$$p=\;\frac{23}{748-388}=6.4\%$$

Solving for the required response rate in Equation 4 yielded a lower operational break-even point of 6.4%. Thus, when applying this exploratory upper-bound scenario, a standard letter invitation strategy would need to achieve a participation rate of 6.4% to match the cost per screened woman of the targeted self-sampling strategy (figure 2).

## Discussion

### Clinical yield

This study evaluated the clinical yield and operational cost thresholds of a targeted HPV self-sampling strategy for long-term non-attenders. Our findings suggest that this strategy can help engage a hard-to-reach population. By evaluating both the direct and cumulative response, we observed the expected direct test returns; additionally, our exploratory upper-bound scenario captured concurrent clinical screening attendances among women who did not return a kit. The clinical effectiveness of the programme can be contextualised by using the NTA and NKD to detect one additional histologically confirmed HSIL+. In the direct response cohort, a NTA_detect_ of 68 and a NKD_detect_ of 418 were required to detect one confirmed HSIL+ case. When accounting for the cumulative response cohort, these operational indicators declined to a NTA_detect_ of 63 and a NKD_detect_ of 353. The overall detection rate of HSIL+ in our study was lower than in earlier Scandinavian studies targeting long-term non-attenders, such as the studies of Elfström *et al* and Aasbø *et al*.^4 17^ While these studies did not explicitly use the same terminology, applying our calculation to their published screening and detection data from their comparable direct-mailing self-sampling arms yields an equivalent cumulative NTA_detect_ of approximately 22 and 26, respectively.

This difference in clinical yield may be driven by several factors, for instance the time of primary HPV screening implementation. Because HPV testing offers higher sensitivity and a higher negative predictive value compared with cytology, a population subjected to primary HPV screening over an extended period probably has a decreased prevalence of precancerous lesions.^18–20^ Prolonged systematic clearance is likely to result in a higher NTA today compared with the past. Furthermore, the variations in NTA and NKD illustrate the expected differences between routine clinical implementation and controlled study environments. The evaluations by Elfström *et al* and Aasbø *et al* were conducted as randomised studies and trials.^4 17^ Trial designs typically enable real-time cleaning of the study population, in contrast to a real-world setting. Consequently, the inclusion of unreachable or ineligible individuals can dilute the overall results, reflecting the well-documented efficacy-effectiveness gap commonly seen when interventions move from trials to routine practice.^21^

NKD_detect_ provides insight into the logistical workload and real-world effectiveness of the ‘send-to-all’ outreach strategy. In our study, the NKD was 418 per detected HSIL+ in the direct response cohort, which decreased to 353 when including the cumulative response. The gap between the NTA_detect_ (63-68) and the NKD_detect_ (353–418) illustrates that the vast majority of dispatched kits are never returned.

### Resource utilisation

Adopting a cumulative response perspective slightly increases the average cost per screened woman from 2,031 SEK (€183) in the direct response cohort to 2,045 SEK (€185). While the inclusion of clinical visits introduces a higher unit cost, it is partially balanced because the upfront costs of the unreturned kits are now distributed across a larger total number of participating women. However, the cost of disease detection improved. Specifically, the average cost per detected HSIL+ decreased from 134,103 SEK (€12,114) to 126,068 SEK (€11,388). This indicates the potential clinical value in this exploratory upper-bound scenario by capturing additional high-grade lesions among women attending clinical screening.

### DTA and the economic break-even point

Our application of a DTA aligns with the decision-analytic principle that identifying the specific threshold values required to alter a decision under uncertainty may be more useful for decision makers than relying on speculative or arbitrary parameter assumptions.^16^ While probabilistic sensitivity analyses are often advocated to capture joint parameter uncertainty, they are primarily necessary when economic models exhibit non-linear relationships. Because our break-even calculation relies on a relationship between established unit costs and the unknown participation rate, a deterministic approach avoids the introduction of arbitrary probability distributions. This approach allows healthcare planners to judge from operational experience whether the required threshold values are plausible or unlikely to occur within a hard-to-reach population.

To contextualise the operational break-even points identified in our threshold analysis (9.5% for the direct response and 6.4% for the cumulative response cohort), it is helpful to consider studies in which standard invitation letters were sent to long-term non-attenders. Previous Scandinavian trials indicate that engaging this specific demographic through traditional standard letter invitations is challenging. For instance, the earlier mentioned Swedish study by Elfström *et al* reported a participation rate of 1.7% in their group of long-term non-attenders receiving standard annual invitation letters, in contrast to 18.7% participation in the group that were sent a kit.^4^ Similarly, the Norwegian study by Aasbø *et al* noted that participation following standard invitation letters declined progressively as the time since the last screening increased; participation was 8.7% among those unscreened for 10–15 years, dropping to 4.0% for those unscreened for 16–28 years and reaching 1.9% among the subgroup of women who had never been screened.^17^ Other Swedish regional trials have reported higher participation rates for standard letter invitations, such as 7.0% in Östergötland (Lilliecreutz *et al*)^5^ and 10.6% in Western Sweden (Broberg *et al*).^22^ Comparing these historical figures to our findings demonstrates that our calculated thresholds (6.4%–9.5%) sit at the upper end of what standard invitations generally achieve in this demographic. When accounting for the exploratory cumulative cohort, the break-even point for standard invitations drops to 6.4% due to the added unit costs of clinical visits. Although historical standard letter participation can exceed this lower 6.4% threshold, the self-sampling strategy presents a clinical advantage. While the cumulative scenario slightly increases the average cost per screened woman, the overall diagnostic yield improves, ultimately lowering the average cost required to detect one additional HSIL+ case.

### Strengths and limitations

A key strength of this study is its population-based, real-world design, evaluating the routine implementation of a targeted self-sampling strategy. A methodological limitation of the threshold analysis is its strict focus on the screening phase, evaluating effectiveness only up to the point of a screening result. Consequently, the economic model does not account for subsequent follow-up costs, such as colposcopies and histological confirmations, nor does it address the comparative clinical utility and sensitivity differences between the two collection methods (self-samples vs clinician-collected samples). Downstream resource allocation and clinical yield are instead captured and reported descriptively. Furthermore, our analysis does not encompass the long-term health economic savings generated by identifying HSIL cases prior to their progression to manifest cervical cancer. Preventing invasive disease inherently averts substantial future costs for both the healthcare system and the individual, including complex oncological treatments, prolonged sick leave and loss of productivity.

Regarding the cumulative response cohort, it is possible that receiving a physical self-sampling kit may have acted as an additional reminder or a nudge, eventually encouraging a subset of women to attend clinical screening. Conversely, some of these clinical attendances might have occurred independently of the self-sampling send-out. Because this is a registry-based study, we lack data on the clinical indications for these visits; therefore, we cannot determine whether some women attended clinical screening due to the onset of symptoms rather than the intervention itself.

Finally, it remains unknown whether the intervention fosters long-term adherence. While we observed instances in our data where long-term non-attenders responded to the self-test and subsequently returned for regular clinical screening, we have not formally quantified the extent of this pattern. It is possible that successfully completing a self-test or re-engaging with the screening programme lowers the threshold for these women to continue participating in future screening rounds. This potential long-term behavioural effect of the kit dispatch remains a highly relevant factor for future research.

## Conclusions

Implementing a targeted HPV self-sampling strategy for long-term non-attenders successfully identifies high-grade cervical lesions within a hard-to-reach population. Our DTA suggests that, from a strictly operational perspective, direct mailing of self-sampling kits is a resource-efficient alternative to standard letter invitations for this specific demographic. Our analysis establishes operational break-even points requiring a standard invitation response rate of 6.4%–9.5% to achieve short-term cost-neutrality per screened woman against self-sampling send-outs. Based on previous research, such participation rates are difficult to achieve using standard invitation letters alone. However, these findings should be interpreted as operational thresholds rather than a full economic evaluation, and future research incorporating long-term health outcomes is required to confirm comprehensive cost-effectiveness.

## Supplementary material

10.1136/bmjopen-2026-125034 online supplemental file 1

## Data Availability

All data relevant to the study are included in the article or uploaded as supplementary information.
