# Supplementary material for: Clinical yield and operational cost-threshold analysis of HPV self-sampling send-outs for long-term non-attenders in cervical cancer screening: a population-based, real-world, registry study in Sweden
Source: BMJ Open. 2026 Oct 22;16(10):e125034. doi: 10.1136/bmjopen-2026-125034 (PMC13711516; doi:10.1136/bmjopen-2026-125034)
Supplement: Supplementary file 1 [file bmjopen-2026-125034.reviewer_comments.pdf]

PEER REVIEW HISTORY

BMJ Open publishes all reviews undertaken for accepted manuscripts. Reviewers are asked to complete a checklist review form and are provided with free text boxes to elaborate on their assessment. These free text comments are reproduced below.

ARTICLE DETAILS

Title (Provisional)

Clinical yield and operational cost-threshold analysis of HPV self-sampling send-outs for long-term non-attenders in cervical cancer screening: A population-based, real-world, registry study in Sweden

Authors

Fridljung, Jasmine; Bergengren, Lovisa; Lillsunde-Larsson, Gabriella; Ryen, Linda

---

VERSION 1 - REVIEW

**Reviewer**	**1**
**Name**	**Kumari, Sarita**
**Affiliation**	**Max Institute of Cancer Care, Gynaecologic Oncology**
**Date**	**30-Jul-2026**
**COI**	**None**

The study is well designed and relevant real-world study addressing a critical gap in cervical cancer screening. i.e. long-term non-attenders. The integration of clinical yield with economic evaluation is a major strength and aligns well with public health priorities and WHO elimination goals. However, I have a few comments:

1) No direct comparison with standard invitation arm/historical control group could lead to a bias in results. Authors could include benchmark participation rates from literature or registry data.

2) Screening uptake may be symptom-driven - is there any possibility of determing the proportion of those cases?

3) Attribution of “Spill-over Effect” could be due to routine invitations, symptoms or external awareness campaigns

Is there a way to address this bias?

4) Authors could highlight differences in mailing cost, laboratory cost or workforce cost compared to routine screening.

5) Could authors identify any subgroup differences leading to low participation rate (1,826/11,283 kits)?

6) Outcome Measure may include: CIN2 vs CIN3 breakdown, Cancer detection rate and HPV positivity rate.

7) What was the accuracy of follow up of positive HPV cases? Rates of loss to follow up?

Reviewer 2

Name Karibayeva, Indira

Affiliation Georgia Southern University, Department of Health Policy and Community Health

Date 06-Aug-2026

COI None

The study provides useful real-world descriptive information, but several methodological issues require major revision. First, the abstract and conclusions overstate what can be inferred from the uncontrolled registry design. The additional 190 clinical attendances among women who did not return a kit cannot be confidently attributed to the self-sampling dispatch because routine screening invitations may have continued, clinical indications were unavailable and no control group or counterfactual comparison was used. The cumulative cohort should therefore be presented as an exploratory or upper-bound scenario rather than as evidence of a behavioural spill-over effect.

Second, the units of analysis require clarification. The manuscript alternates between unique women, dispatched kits, returned kits and screening episodes. Direct NNS is based on analysed kits, whereas Table 2 reports screened women, and cumulative NNS combines analysed kits with additional clinically screened women. Similarly, NNI is based on 11,283 kit dispatches rather than 9,123 unique invited women. The authors should either recalculate person-level measures or rename them as kit dispatches or analysed tests per detected HSIL+ case.

Third, the economic analysis should be described as an operational cost-threshold analysis rather than a cost-effectiveness evaluation. Cost per screened woman does not capture comparative test performance, follow-up completion, downstream diagnostic consequences or longer-term health outcomes. The comparator must also be costed symmetrically, including any standard invitations sent concurrently with the self-sampling kits. The interpretation of the 8.3% threshold should be corrected: this lower threshold means that standard invitations need a lower participation rate to become cost-equivalent, and therefore it does not strengthen the economic case for self-sampling.

Finally, uncertainty is insufficiently examined. Confidence intervals should be reported for key clinical outcomes, and scenario analyses should vary unit costs, repeated dispatch

assumptions and the proportion of additional clinical attendances attributable to the intervention. The effect of incomplete or delayed follow-up among HPV-positive women should also be evaluated.

VERSION 1 - AUTHOR RESPONSE

Reviewer : 1<br>Dr. Sarita Kumari, Max Institute of Cancer Care	
Reviewer comments	Our response
The study is well designed and relevant real-world study addressing a critical gap in cervical cancer screening. i.e. long-term non-attenders. The integration of clinical yield with economic evaluation is a major strength and aligns well with public health priorities and WHO elimination goals. However, I have a few comments.	We would like to express our gratitude for your review and encouraging opening remarks. We are delighted that you found the study well-designed. Your comments have been valuable in helping us improve the manuscript. Please find our point-by-point responses below.
No direct comparison with standard invitation arm/historical control group could lead to a bias in results. Authors could include benchmark participation rates from literature or registry data.	We appreciate the reviewer highlighting this important methodological aspect. We agree that the lack of a direct historical control group is a limitation, which we have actively addressed in the manuscript in the following ways:<br>- The lack of historical control data for this specific subgroup within standard administrative systems is explicitly acknowledged as a methodological challenge in the Introduction (Page 3, Lines 34–45 and Page 4, Lines 1-2).<br>- To compensate for this and accurately contextualise our operational break-even points, we have already included benchmark participation rates from comparable Scandinavian trials in the discussion (Page 10, Lines 33-44; Page 11, Lines 1-10).
Screening uptake may be symptom-driven - is there any possibility of determining the proportion of those cases?	We agree with the reviewer that the clinical visits, could be symptom-driven. Unfortunately, it is not possible to determine the proportion of these cases within the current study design. Because this is a population-based study relying on regional laboratory and administrative registries, we only have data on dispatched kits, returned kits, and registered clinical screening visits. We do not have access to patient medical records, which would be required to capture patient-reported symptoms or the specific indication for a visit. Furthermore, when these women attend a clinical follow-up, an indicated test is performed regardless of whether the visit was prompted by the intervention or by the onset of symptoms. We have ensured this limitation is clearly stated in the manuscript (Page 2, Lines 10-11 and Page 11, Lines 24-30)
Attribution of “Spill-over Effect” could be due to routine invitations, symptoms or external awareness campaigns. Is there a way to address this bias?	Because this is a retrospective registry-based study, we unfortunately lack the data on clinical indications required to isolate the intervention's effect from concurrent routine invitations or symptom-driven attendances. While we aimed to describe it as a *potential* spill-over effect we acknowledge that the terminology used was too definitive given the study design. To increase transparency, we have substantially revised the manuscript. We have removed the term "spill-over effect" throughout the text and now strictly describe

	the cumulative cohort as an "exploratory upper-bound scenario."
Authors could highlight differences in mailing cost, laboratory cost or workforce cost compared to routine screening.	A breakdown highlighting the differences in mailing costs, laboratory costs, and workforce costs is provided in "Appendix B: Specification of unit costs and resource components". To make this more prominent for the reader, we have added a clearer reference to Appendix B in the main text (Page 6, Lines 26-27).
Could authors identify any subgroup differences leading to low participation rate (1,826/11,283 kits)?	For the specific demographic of long-term non-attenders, this return rate could represent an improvement over standard letter invitation. The fact that our participation is lower than in previous controlled trials could reflect a well-documented "efficacy-effectiveness gap" discussed in Page 10, Lines 4-7.<br>We agree that identifying subgroup differences (e.g., by age or socioeconomic status) among the non-responders would be highly valuable. However, our ethical approval was strictly limited to the clinical parameters and did not allow for the retrieval of such demographic variables.
Outcome Measure may include: CIN2 vs CIN3 breakdown, Cancer detection rate and HPV positivity rate	We thank the reviewer for these suggestions. Regarding the CIN2 vs CIN3 breakdown, Sweden has a routine not to distinguish between cervical intraepithelial neoplasia, CIN, 2 and 3. Because of the real world setting of the study there is no data on CIN classification available nor would that impact how women would be followed up. Regarding the cancer detection rate, invasive cancers are included in our primary outcome measure (HSIL+) and are explicitly specified in both the Results section and in Figure 1 (two cases in the direct cohort, one in the cumulative).<br>Regarding the HPV positivity rate, the absolute numbers are presented in Figure 1 and the text. To further highlight this outcome measure, we have now added the specific positivity rate directly into Table 2 (Page 8).
What was the accuracy of follow up of positive HPV cases? Rates of loss to follow up?	We agree that the accuracy and rate of clinical follow-up are important outcomes. The absolute numbers regarding follow-up for HPV-positive cases are detailed in the study flow chart (Figure 1). Among the 319 unique women with an HPV-positive self-sample, 259 attended a clinical follow-up within one year, 5 women had a delayed follow-up (>1 year), and 55 women had no registered clinical follow-up. To make the rate of loss to follow-up more explicit for the reader, we have now added these specific percentages into Table 2 (Page 8).

Reviewer: 2<br>Dr. Indira Karibayeva, Georgia Southern University	
Reviewer comments	Our response
The study provides useful real-world descriptive information, but several methodological issues require major revision.	We would like to express our gratitude for your review and encouraging opening remark. Your comments have been very valuable in helping us improve the manuscript. Please find our point-by-point responses below.
First, the abstract and conclusions overstate what can be inferred from the uncontrolled registry design. The additional 190 clinical attendances among women who did not	We agree with the reviewer's assessment. While we aimed to describe it as a *potential* spill-over effect we acknowledge that the terminology used was too definitive given the study design. To accurately reflect this, we have revised the

return a kit cannot be confidently attributed to the self-sampling dispatch because routine screening invitations may have continued, clinical indications were unavailable and no control group or counterfactual comparison was used. The cumulative cohort should therefore be presented as an exploratory or upper-bound scenario rather than as evidence of a behavioural spill-over effect.	manuscript throughout. Following the reviewer's suggestion, we now frame the cumulative cohort as an "exploratory upper-bound scenario" rather than a potential behavioural spill-over effect.
Second, the units of analysis require clarification. The manuscript alternates between unique women, dispatched kits, returned kits and screening episodes. Direct NNS is based on analysed kits, whereas Table 2 reports screened women, and cumulative NNS combines analysed kits with additional clinically screened women. Similarly, NNI is based on 11,283 kit dispatches rather than 9,123 unique invited women. The authors should either recalculate person-level measures or rename them as kit dispatches or analysed tests per detected HSIL+ case.	As the reviewer correctly notes, some women received multiple kits during the study period (11,283 kits dispatched to 9,123 unique women). Because our economic threshold analysis is built on direct resource utilisation (where every dispatched kit and analysed test carries a cost, regardless of whether it is the first or second attempt for a specific woman), it is necessary for us to retain the event-level calculations. However, we fully agree that the terminology must accurately reflect the unit of analysis. To resolve this and avoid misinterpretations, we have adopted the reviewer's suggestion to rename the metrics. Throughout the manuscript, including the abstract, we have replaced NNS and NNI with the following operational terms:<br>- NTA (Number of Tests Analysed per detected HSIL+ case) replaces NNS.<br>- NKD (Number of Kits Dispatched per detected HSIL+ case) replaces NNI.<br>We believe this clarification improves the methodological transparency.
Third, the economic analysis should be described as an operational cost-threshold analysis rather than a cost-effectiveness evaluation.	We appreciate the reviewer's attention to health economic terminology. As outlined in the Introduction, our study was designed as an alternative to a traditional cost-effectiveness evaluation (such as calculating an ICER) due to the lack of historical control data. We do not claim to present a formal cost-effectiveness analysis. However, we agree that broader terms used in the manuscript, such as "economic effects", might inadvertently imply a full economic evaluation. To ensure clarity we have refined the terminology throughout the manuscript. We now consistently use the terms "operational cost-threshold analysis" and "resource utilisation", and we have updated the manuscript's title accordingly to reflect this.
Cost per screened woman does not capture comparative test performance, follow-up completion, downstream diagnostic consequences or longer-term health outcomes.	We agree with the reviewer's assessment. "Cost per screened woman" is strictly an operational metric. A full economic evaluation with a longer time perspective would be required to capture these parameters. However, as outlined in the Introduction, applying a full-pathway model to this specific, hard-to-reach cohort would rely heavily on speculative assumptions due to the lack of historical baseline data. Therefore, we deliberately restricted our analysis to an operational cost-threshold model focusing solely on the primary screening phase and the observed data. We believe that by renaming the metrics (to NTA/NKD) and clarifying the terminology to "operational cost-threshold analysis" (as suggested in your previous comments), the restricted scope

	of these intermediate metrics is now even more accurately reflected throughout the manuscript.
The comparator must also be costed symmetrically, including any standard invitations sent concurrently with the self-sampling kits.	We highly appreciate the reviewer's sharp methodological observation. Because the self-sampling kit is implemented as an addition to standard invitations, we agree that the administrative burden of concurrent standard invitations must be represented to ensure a symmetrical operational comparison. We have now revised our calculations to account for this. Because exact individual dispatch timestamps for standard invitations are unknown and not perfectly synchronized between the regional laboratory system (handling the kits) and the general screening registry (handling standard invitations), we cannot retrospectively determine exactly how many concurrent standard letters (e.g., 0, 1, or 2) each specific woman received during the study period and after her receiving a self-sampling send out. Therefore, as a pragmatic approximation of this concurrent administrative burden, a single standard letter unit cost was assigned to the absolute non-attending sub-cohort (n=7,150, women who neither returned a self-sampling kit nor attended clinical screening). Women who participated in screening were excluded from this specific cost allocation, as generating a screening result inherently suspends further standard invitations for the current screening cycle. As a result of this modification, the total costs and subsequent operational break-even points have been recalculated throughout the manuscript. We have updated all corresponding values and added a dedicated explanatory paragraph in the Method section and updated Table 1 and Appendix B to transparently display this cost component.
The interpretation of the 8.3% threshold should be corrected: this lower threshold means that standard invitations need a lower participation rate to become cost-equivalent, and therefore it does not strengthen the economic case for self-sampling.	We thank the reviewer for pointing this out. The inclusion of the cumulative exploratory cohort increases the average cost per screened woman (since clinical visits are more expensive than self-sample laboratory analyses), which correspondingly lowers the required break-even participation rate for standard invitations. This indeed means standard invitations need a lower participation rate to become cost equivalent. As suggested, we have revised the final paragraph of the discussion (Page 11, Lines 2-10).
Finally, uncertainty is insufficiently examined. Confidence intervals should be reported for key clinical outcomes, and scenario analyses should vary unit costs, repeated dispatch assumptions and the proportion of additional clinical attendances attributable to the intervention. The effect of incomplete or delayed follow-up among HPV-positive women should also be evaluated.	We appreciate the reviewer's suggestions for strengthening the examination of uncertainty. We have addressed this in the following ways:<br>1. Confidence Intervals<br>We agree that statistical uncertainty should be reported for the clinical proportions. We have now added 95% confidence intervals (calculated using the Wilson score interval method) for the key clinical outcomes: kit return rate, overall participation rate, and HPV-positivity rate. These have been added to Table 2 and detailed in the method section.<br>2. Scenario analyses<br>Because our study is a retrospective evaluation of observed, real-world registry data, we chose not to introduce the arbitrary behavioural assumptions required for predictive scenario analyses.<br>Economic uncertainty: Our Deterministic Threshold Analysis (DTA) inherently acts as a sensitivity analysis. Rather than

	arbitrarily varying unit costs, Figure 2 maps the break-even tipping points across a continuous spectrum of standard participation rates.<br>Incomplete follow-up: The clinical and economic penalties of incomplete follow-up are already captured in our results. Because our outcomes require histologically confirmed HSIL+, women lost to follow-up contribute fully to the operational costs and total analysed tests but yield zero clinical benefit. This naturally penalises the reported NTA, NKD, and average cost per detected case. For transparency, the absolute numbers of absent or delayed follow-ups are now explicitly detailed in Table 2.

VERSION 2 - REVIEW

Reviewer 1

Name Kumari, Sarita

Affiliation Max Institute of Cancer Care, Gynaecologic Oncology

Date 11-Sep-2026

COI

Thank you for addressing the comments

Reviewer 2

Name Karibayeva, Indira

Affiliation Georgia Southern University, Department of Health Policy and Community Health

Date 11-Sep-2026

COI

The manuscript has substantially improved. I would suggest to moderate the economic conclusions and update the CHEERS checklist

VERSION 2 - AUTHOR RESPONSE

Reviewer 1

Reviewer comment:
Thank you for addressing the comments.

Our response:
We sincerely thank the reviewer for the time and constructive feedback provided during this review process.

Reviewer 2

Reviewer comment:
The manuscript has substantially improved. I would suggest to moderate the economic conclusions and update the CHEERS checklist.

Our response:
Thank you for the positive feedback and the additional time you have taken to provide constructive comments.

Following your suggestion, we have further moderated the economic conclusions in both the abstract and in the text following the subheading “conclusions” to emphasize the operational nature of our findings.

Additionally, we have updated the CHEERS checklist to reflect the methodological updates and terminology introduced during the revision process.
