# Supplementary material for: Clinical yield and operational cost-threshold analysis of HPV self-sampling send-outs for long-term non-attenders in cervical cancer screening: a population-based, real-world, registry study in Sweden
Source: BMJ Open. 2026 Oct 22;16(10):e125034. doi: 10.1136/bmjopen-2026-125034 (PMC13711516; doi:10.1136/bmjopen-2026-125034)
Supplement: Supplementary file 2 [file bmjopen-16-10-s001.docx]

## Appendix A: Derivation of the threshold equation

This appendix outlines the mathematical derivation used to determine the break-even participation rate (*p*). This point is defined as the theoretical participation rate at which mailed standard invitations become equal to the observed cost per screened woman in the targeted self-sampling strategy.

- *p:* Theoretical participation rate for standard invitations.
- *N*: Total number of invited women.
- $C_{invite}$: Administrative cost of sending one standard invitation (e.g., postage, printing, administration).
- $C_{test}:$Cost incurred only when a woman attends standard screening (e.g., midwife visit, consumables, and HPV analysis).
- ${AC}_{self\_direct}$: Average cost per screened woman using the self-sampling strategy (fixed, observed value from the study).
- ${AC}_{self\_cumulative}$: Adjusted average cost per screened woman when accounting for the cumulative response cohort (fixed, observed value from the study).
- ${TC}_{inv}$: Total cost of standard invitation strategy.
- ${AC}_{inv}$: Average cost per screened woman using standard invitations.

The total cost of a standard invitation strategy to a cohort of long-term non-attenders includes the fixed administrative cost for all invited women, plus the variable clinical cost for the subset of women who actually attend screening:

$${TC}_{inv}=N\cdot C_{invite}+N\cdot p\cdot C_{test}$$

To calculate the average cost per screened woman for standard invitations, the total cost is divided by the number of women who participate:

$${AC}_{inv}= \frac{N\cdot C_{invite}+N\cdot p\cdot C_{test}}{N\cdot p}$$

The break-even participation rate is identified at the exact point where the average cost per screened woman using standard invitations equals the observed average cost of the self-sampling strategy:

$$\frac{C_{invite}}{p}+ C_{test}= {AC}_{self}$$

Isolating the theoretical participation rate provides the threshold equations:

$p= \frac{C_{invite}}{{AC}_{self\_direct}-C_{test}} (Eq. 3)$	$p= \frac{C_{invite}}{{AC}_{self\_cumulative}-C_{test}} (Eq. 4)$

## Appendix B: Specification of unit costs and resource components

| Resource category | Cost component description | Component cost (SEK) | Total unit cost (SEK) |
| --- | --- | --- | --- |
| *Outreach and administration* |  |  |  |
| Dispatched self-sampling kits | Kit, packaging, return postage, and handling | 45 | 45 |
| Standard invitation letters | Postage and handling | 23 | 23 |
| *Screening phase* |  |  |  |
| HPV analysis of returned kits | Aptima HPV analysis | 246 | 246 |
| Midwife screening visits | Midwife salary (based on  a 17-minute visit) | 105 |  |
|  | Consumables | 37 |  |
|  | Aptima HPV analysis | 246 | 388 |
| *Clinical follow-up* |  |  |  |
| Colposcopies | Colposcopy procedure | 7,096 |  |
|  | Cytology analysis | 481 |  |
|  | Aptima HPV analysis | 246 | 7,823 |
| Genotyping of HPV-positive tests | ALLPLEX HPV  genotyping analysis | 915 | 915 |
| Histopathological analysis | Histological examination  of biopsies | 1,472 | 1,472 |
| Notes: The procedure cost for colposcopy is based on the regional cost per patient database (KPP) from 2024. All remaining unit costs were retrieved from regional administrative registries at the 2025 price level. To ensure a symmetrical cost comparison in the threshold analysis, we accounted for concurrent standard invitations sent by the general screening programme. Specifically, a standard letter unit cost (23 SEK) was assigned to the 7,150 women who neither returned a self-sampling kit nor attended clinical screening. | | | |
